# Dataset of surface refractivity in Southeast, Nigeria

**DOI:** 10.1016/j.dib.2017.11.052

**Published:** 2017-11-20

**Authors:** Sayo A. Akinwumi, Temidayo V. Omotosho, Oluwole A. Odetunmibi

**Affiliations:** a Department of Physics, Covenant University, Ota, Nigeria; b Department of Mathematics, Covenant University, Ota, Nigeria

**Keywords:** Refractivity, Water vapour density, Communication, Radiowave propagation

## Abstract

In this data article, analysis of surface refractivity and water vapour density in Southeast, Nigeria were reported. The meteorological data were collected for the period of 39 years between 1973 and 2012 from National Oceanic and Atmospheric Administration (NOAA) Climatology Centre. Five locations considered in the study area includes: Enugu, Onitsha, Abakaliki, Aba and Ihiala. Descriptive statistics were used to show an increase in monthly variation of refractivity of about 299.8 N units at Enugu in January to peak value of about 385.81 N units at Abakaliki in May. Hence, the seasonal variation for South East indicate maximum value within the months of March to May in the rainy season and a minimum value around December to February which is the dry season. The results from this data will help engineers in proper design and planning of radiowave propagation and satellite communication systems in southeastern, Nigeria.

**Specifications Table**

**Table:** 

Subject area	*Meteorology and Atmospheric environment*
More specific subject area	*Satellite Communication, Radiowave propagation, Radio Science*
Type of data	*Table and figure*
How data was acquired	*Secondary data*
Data format	*Raw and analyzed*
Experimental factors	*Data Obtained from National Oceanic and Atmospheric Administration (NOAA) Climatology Centre*
Experimental features	*Computational Analysis: Contingency Tables*
Data source location	*Data Obtained from National Oceanic and Atmospheric Administration (NOAA) Climatology Centre, USAF*
Data accessibility	*All the data are in this article as a supplementary file*

**Value of the data**

• The data could be useful for government in understanding of radio propagation within or around the lower atmosphere in the southeast region of Nigeria.
• The database could provide insights of radio refractivity and water vapour density for the five locations.
• The dataset will help engineers in siting good antenna reception at ground level for AM, FM, VHF, UHF bands in Nigeria.
• The data will be useful in understanding of the refractive index structure of the atmosphere through which the waves travel.

## 1 Data

The meteorological data for this article were obtained from National Oceanic and Atmospheric Administration (NOAA) Climatology center for the period of about thirty-nine years from 1973 through 2012 for five locations within southeast, Nigeria. The data input parameters such as pressure, temperature, and relative humidity were used for the calculation of surface radio refractivity (N) for all the location. The meteorological data assembled were based on one-minute to produce the daily average data and consequently to acquire the monthly. Therefore, the monthly means of the measurements, over the thirty-nine years is a good characteristic of the seasonal behavior of surface radio refractivity as revealed in Table 1a, Table 1b, Table 1c, Table 1d, Table 1e. The descriptive statistics summaries of the surface refractivity are presented tables. While, bar charts for the refractivity distribution are presented in figures.

**Table 1a:** Monthly refractivity values from Enugu State.

**Month**	**Jan**	**Feb**	**Mar**	**Apr**	**May**	**Jun**	**Jul**	**Aug**	**Sep**	**Oct**	**Nov**	**Dec**
**Refractivity***N units*	299.8	338.7	368.02	378.69	375.7	374.27	375.5	374.58	376.91	376.71	349.7	335.9

**Table 1b:** Monthly refractivity values from Anambra State.

**Month**	**Jan**	**Feb**	**Mar**	**Apr**	**May**	**Jun**	**Jul**	**Aug**	**Sep**	**Oct**	**Nov**	**Dec**
**Refractivity***N units*	373.3	381.5	384.63	385.17	384.6	381.07	374.51	374.29	377.99	379.06	379.9	373.6

**Table 1c:** Monthly refractivity values from Ebonyi State.

**Month**	**Jan**	**Feb**	**Mar**	**Apr**	**May**	**Jun**	**Jul**	**Aug**	**Sep**	**Oct**	**Nov**	**Dec**
**Refractivity***N units*	370.6	380.3	383.11	385.51	385.8	382.41	379.05	378.44	379.8	380.57	380.9	372.2

**Table 1d:** Monthly refractivity values from Abia State.

**Month**	**Jan**	**Feb**	**Mar**	**Apr**	**May**	**Jun**	**Jul**	**Aug**	**Sep**	**Oct**	**Nov**	**Dec**
**Refractivity***N units*	371.9	376.5	378.88	379.62	379.8	376.83	372.3	370.78	373.85	374.45	376.1	371.9

**Table 1e:** Monthly refractivity values from Imo State.

**Month**	**Jan**	**Feb**	**Mar**	**Apr**	**May**	**Jun**	**Jul**	**Aug**	**Sep**	**Oct**	**Nov**	**Dec**
**Refractivity***N units*	377.3	381.5	384.01	384.49	384.4	380.07	374.83	372.86	376.85	378.36	378.7	376.7

Radio refractivity, *N*, depends on meteorological parameters such as the pressure *P* (mbar), the absolute air temperature *T* (K), and the vapour pressure e (mbar) as given in Eq. (1).

(1) $$N=\frac{77.6P}{T}+3.73\times 10^{5}\frac{e}{T^{2}}=N_{\mathrm{dry}}+N_{\mathrm{wet}}\left(N-\mathrm{units}\right)$$

$N_{\mathrm{dry}}$ and $N_{\mathrm{wet}}$ are frequently denoted as dry and wet terms of atmospheric radio refractivity, respectively.

(2) $${\mathrm{where}:\;N}_{\mathrm{dry}}=\frac{77.6P}{T}$$

and

(3) $$N_{\mathrm{wet}}=3.73\times 10^{5}\frac{e}{T^{2}}$$

The nature and usefulness of the data entails that it can be analyzed using different statistics techniques like ordinary least square regression analysis, simple correlation, multiple correlation analysis, analysis of variance, factor analysis and principal component analysis just to mention few.

### 1.1 The summary statistics of the data from Enugu state

**Fig. 1:**
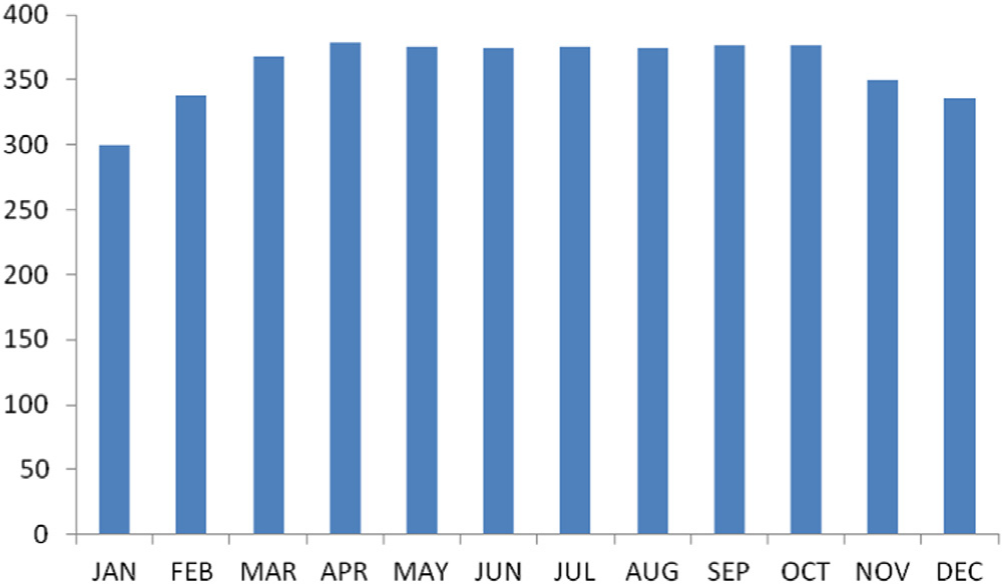
The bar chart showing the monthly refractivity for Enugu state.

The summary statistics of the data collected from Enugu state is presented in the Table 2 below. The data was also presented in a bar chart in Fig. 1. The bar chart is a representation of the descriptive statistics which revealed the level of radio refractivity recorded monthly for the state.

**Table 2:** Summary statistics of the Enugu state Refractivity data.

Mean	360.3698
Std. Error of Mean	7.09456
Median	374.4201
Mode	299.80^a^
Std. Deviation	24.57628
Variance	603.994
Skewness	-1.618
Std. Error of Skewness	.637
Kurtosis	2.230
Std. Error of Kurtosis	1.232
Range	78.89
Minimum	299.80
Maximum	378.69

### 1.2 The summary statistics of the data from Anambra state

**Fig. 2:**
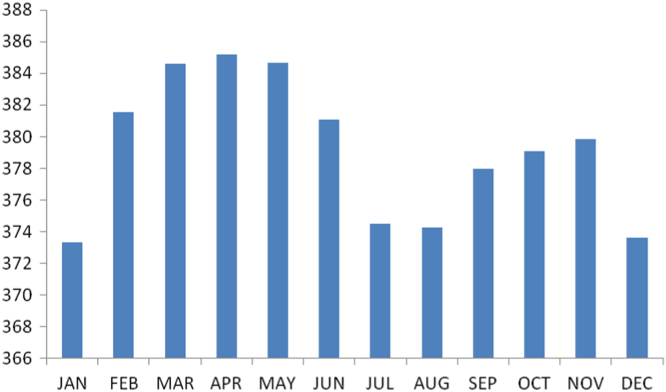
The bar chart showing the monthly refractivity for Anambra state.

The summary statistics of the data collected from Anambra state is presented in the Table 3 below. The data was also presented in a bar chart in Fig. 2. The bar chart is a representation of the descriptive statistics which revealed the level of radio refractivity recorded monthly for the state.

**Table 3:** Summary statistics of the Anambra state Refractivity data.

**Statistics**	**Value**
Mean	379.1399
Std. Error of Mean	1.28354
Median	379.4653
Mode	373.30^a^
Std. Deviation	4.44630
Variance	19.770
Skewness	−.012
Std. Error of Skewness	.637
Kurtosis	−1.498
Std. Error of Kurtosis	1.232
Range	11.87
Minimum	373.30
Maximum	385.17
Sum	4549.68

### 1.3 The summary statistics of the data from Ebonyi state

**Fig. 3:**
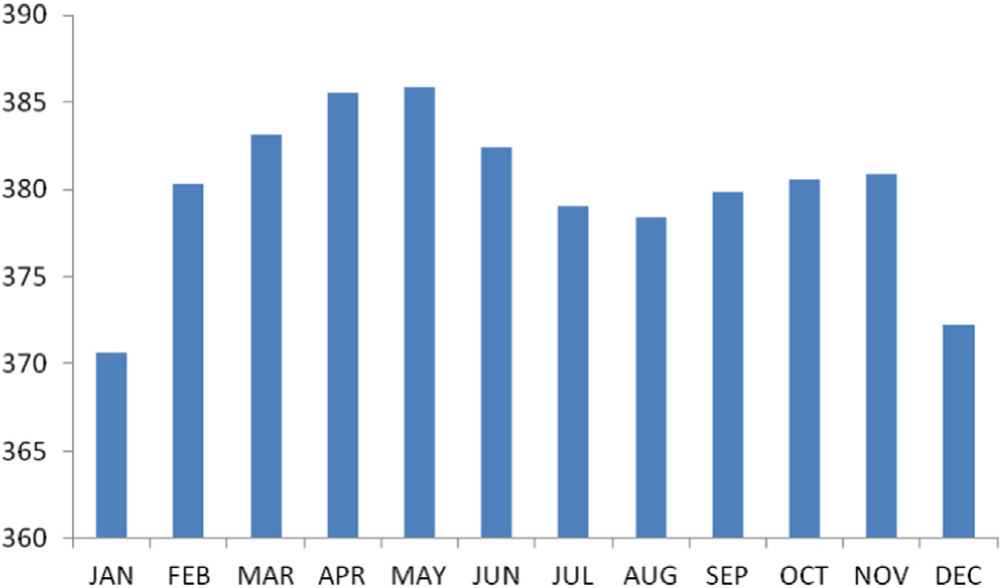
The bar chart showing the monthly refractivity for Ebonyi state.

The summary statistics of the data collected from Ebonyi state is presented in the Table 4 below. The data was also presented in a bar chart in Fig. 3. The bar chart is a representation of the descriptive statistics which revealed the level of radio refractivity recorded monthly for the state.

**Table 4:** Summary statistics of the Ebonyi state refractivity data.

**Statistics**	**Value**
Mean	379.8946
Std. Error of Mean	1.32606
Median	380.4472
Mode	370.63^a^
Std. Deviation	4.59359
Variance	21.101
Skewness	−.917
Std. Error of Skewness	.637
Kurtosis	.633
Std. Error of Kurtosis	1.232
Range	15.18
Minimum	370.63
Maximum	385.81
Sum	4558.74

### 1.4 The summary statistics of the data from Abia state

**Fig. 4:**
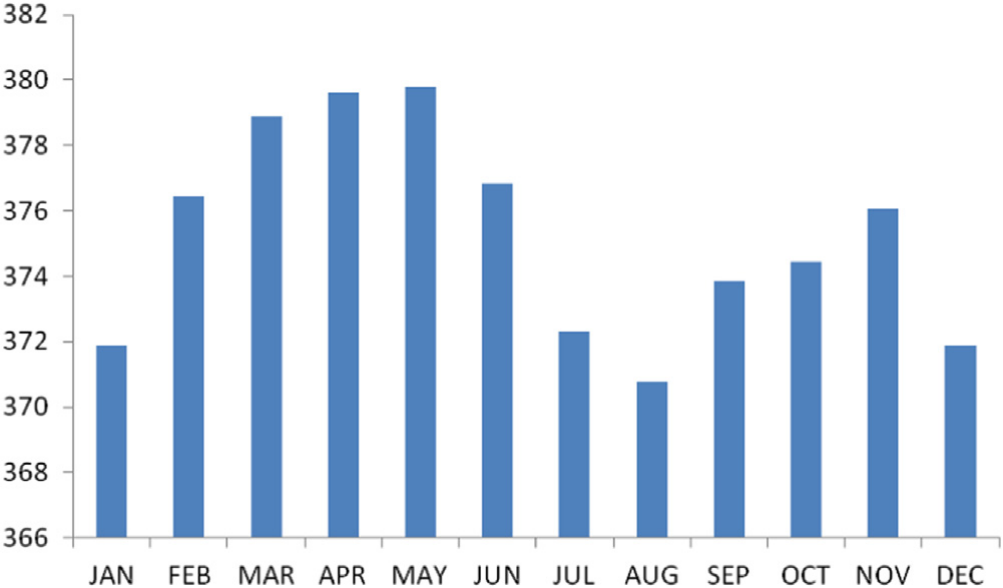
The bar chart showing the monthly refractivity for Abia state.

The summary statistics of the data collected from Abia state is presented in the Table 5 below. The data was also presented in a bar chart in Fig. 4. The bar chart is a representation of the descriptive statistics which revealed the level of radio refractivity recorded monthly for the state.

**Table 5:** Summary statistics of the Abia state refractivity data.

**Statistics**	**Value**
Mean	375.2337
Std. Error of Mean	.91972
Median	375.2667
Mode	370.78^a^
Std. Deviation	3.18600
Variance	10.151
Skewness	.142
Std. Error of Skewness	.637
Kurtosis	−1.409
Std. Error of Kurtosis	1.232
Range	9.02
Minimum	370.78
Maximum	379.80
Sum	4502.80

### 1.5 The summary statistics of the data from Imo state

**Fig. 5:**
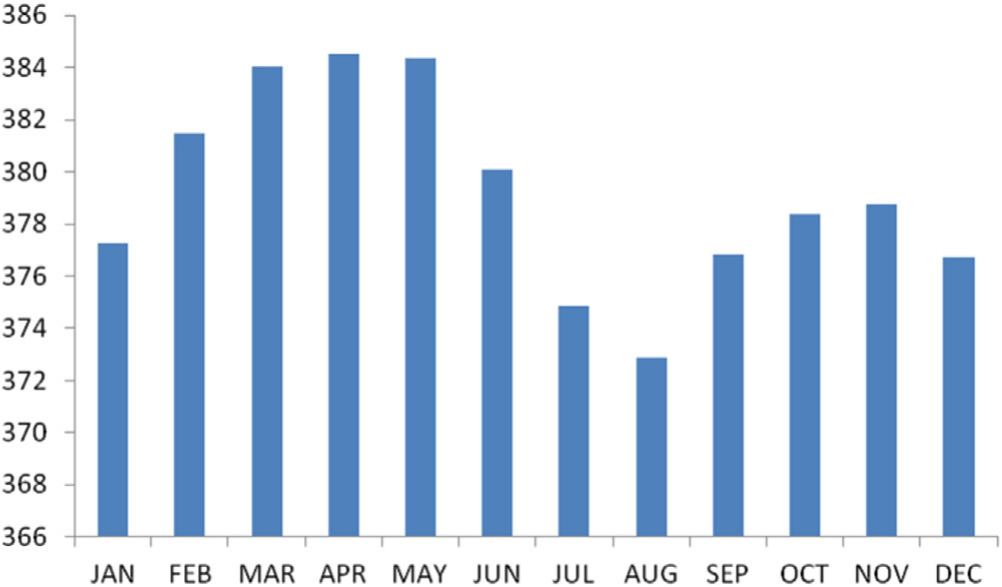
The bar chart showing the monthly refractivity for Imo state.

The summary statistics of the data collected from Imo state is presented in the Table 6 below. The data was also presented in a bar chart in Fig. 5. The bar chart is a representation of the descriptive statistics which revealed the level of radio refractivity recorded monthly for the state.

**Table 6:** Summary statistics of the Abia state refractivity data.

**Statistics**	**Value**
Mean	379.1671
Std. Error of Mean	1.09950
Median	378.5416
Mode	372.86^a^
Std. Deviation	3.80877
Variance	14.507
Skewness	.100
Std. Error of Skewness	.637
Kurtosis	−.938
Std. Error of Kurtosis	1.232
Range	11.64
Minimum	372.86
Maximum	384.49
Sum	4550.01

## 2 Materials and methods

Several researches have been conducted on surface radio refractivity in Nigeria [1], [2], [3], [4], [5], [6], [7], [8], [9], [10], [11], [12], [13]. However, this work is relevant with a focus on southeast zone, Nigeria where few research has been concluded. Hence, formed the uniqueness of this data and the analysis. Similar statistical tools on refractivity were applied by [14], [15]. Radiosonde data for at least 39 years between 1973 and 2012 for 5 stations within Southeast Nigeria were utilized for the computation. It was launched from National Oceanic and Atmospheric Administration (NOAA) Climatology center based in United State of America (USA). The variables contained in the meteorology data such as pressure, temperature, and relative humidity were used as input parameters for the outcome of this article.
